# Financial impact of the change in the vulnerability profile of More Doctors Program

**DOI:** 10.11606/s1518-8787.2020054002156

**Published:** 2020-12-04

**Authors:** Denise de Fátima Barros Cavalcante, Carolina Vitti Domingues, Diego Roberto Meloni, Frederico Machado de Almeida, Livia Fernandes Probst, Yuri Wanderley Cavalcanti, Marcelo de Castro Meneghim, Antonio Carlos Pereira

**Affiliations:** I Universidade Estadual de Campinas Faculdade de Odontologia de Piracicaba PiracicabaSP Brasil Universidade Estadual de Campinas. Faculdade de Odontologia de Piracicaba. Pós-Doutoranda em Odontologia em Saúde Coletiva. Piracicaba, SP, Brasil; II Universidade Estadual de Campinas Faculdade de Odontologia de Piracicaba Programa de Pós-Graduação em Odontologia PiracicabaSP Brasil Universidade Estadual de Campinas. Faculdade de Odontologia de Piracicaba. Programa de Pós-Graduação em Odontologia. Piracicaba, SP, Brasil; III Universidade de São Paulo Faculdade de Medicina de Ribeirão Preto Programa de Pós-Graduação em Saúde Pública Ribeirão PretoSP Brasil Universidade de São Paulo. Faculdade de Medicina de Ribeirão Preto. Programa de Pós-Graduação em Saúde Pública. Ribeirão Preto, SP, Brasil; IV Faculdade de Ciências Médicas da Santa Casa de São Paulo São PauloSP Brasil Faculdade de Ciências Médicas da Santa Casa de São Paulo. São Paulo, SP, Brasil; V Universidade Federal de Mato Grosso do Sul Faculdade de Odontologia de Campo Grande Departamento de Saúde Coletiva Campo GrandeMS Brasil Universidade Federal de Mato Grosso do Sul. Faculdade de Odontologia de Campo Grande. Departamento de Saúde Coletiva. Campo Grande, MS, Brasil; VI Universidade Federal da Paraíba Centro de Ciências da Saúde Departamento de Odontologia Social Joao PessoaPB Brasil Universidade Federal da Paraíba. Centro de Ciências da Saúde. Departamento de Odontologia Social. Joao Pessoa, PB, Brasil; VII Universidade Estadual de Campinas Faculdade de Odontologia de Piracicaba Departamento de Odontologia Social PiracicabaSP Brasil Universidade Estadual de Campinas. Faculdade de Odontologia de Piracicaba. Departamento de Odontologia Social. Piracicaba, SP, Brasil

**Keywords:** Foreign Medical Graduates, supply & distribution, Health Consortia, Health Expenditures, Program Evaluation, economics

## Abstract

**OBJECTIVE:**

To estimate the flow of professionals and the financial impact of the *Programa Mais Médicos para o Brasil* (PMMB – More Doctors for Brazil Program) within the More Doctors Program (MDP) for the Brazilian Ministry of Health and the participating municipalities of the state of São Paulo, from January 2019 to March 2022.

**METHODS:**

A financial impact study was conducted in the state of São Paulo based on public secondary databases. The number of PMMB vacancies per municipality, of physicians and vulnerability profiles were described to measure the loss of replacement of professionals in the period.

**RESULTS:**

In the specified period, the number of PMMB physicians in participating cities will decrease from 2,533 to 320, and the number of participating municipalities from 373 to 86. The municipalities that will need to replace the physicians will have a financial impact of R$ 929,487,904.77 (with sensitivity analysis, ranging from R$ 650,641,533.34 to R$ 1,208,334,276.20).

**CONCLUSION:**

The change of vulnerability methodology adopted for the PMMB will represent serious consequences, that is, less population assistance and high financial impact for the municipalities of the state of São Paulo in a scenario of budget limitations.

## INTRODUCTION

The whole context of changing the collective view present in the framework of the implementation and expansion of the *Política Nacional de Atenção Básica* (PNAB – National Primary Care Policy), from the Unified Health System (SUS), brought concrete demands both in professional training and work management for health policies, being clearly identified in the Brazilian Ministry of Health (MH), in the state and municipal departments^1,2^.

The SUS corroborated the need to implement a new policy that would be able to solve the lack of medical professionals, the deficit of access due to their poor distribution and the quality of primary care, which reflected the lack of improvements in training for the public service. Following this direction, a survey by the *Instituto de Pesquisa Econômica Aplicada* (IPEA – Institute of Applied Economic Research), in 2011, revealed the lack of doctors as the main problem of the SUS for 58.1% of Brazilians, whereas the campaign “where are the doctors?”, conducted by the *Frente Nacional de Prefeitos* (FNP – National Front of Mayors) in Brasilia, in 2013, demonstrated the dissatisfaction of managers with the fixation of medical professionals in their territories^3^.

Thus, the implementation of the More Doctors Program (MDP) in 2013 (Law No. 12,871/2013) meant not only the creation of a qualification policy and budgetary investment of the federal government in primary care in Brazilian municipalities, but also the recognition of these demands in the institutional political context. The MDP is composed of three axes: (a) expansion and improvement of the infrastructure of health units; (B) emergency provision of doctors for unassisted areas; and (c) training of human resources for SUS^4^. Regarding provision, doctors graduated from Brazilian and foreign higher education institutions adhered to the *Programa Mais Médicos para o Brasil* (PMMB – More Doctors for Brazil Program), the specific interest of our study.

Since the beginning of the PMMB, the MH interfered directly with the provision for these municipalities by calling physicians registered in the Regional Councils of Medicine (CRM), individual exchange students (Brazilians and foreigners trained abroad) and Cuban doctors (these in cooperation with the Pan-American Health Organization – PAHO), so that recruitments were conducted in phases, according to the order cited, until the filling of all vacancies. Thus, the MH was the protagonist in the allocation, payment of scholarships, promotion and expansion of medical education change^5^.

Initially, in 2013, the rules for adherence of priority municipalities to the emergency provision of the PMMB were measured by vulnerability indicators based on GDP, health plan coverage, the number of residents in rural areas, extreme poverty, the percentage of Bolsa Família beneficiaries, the hours worked by physicians in primary care, the percentage of beds per thousand inhabitants and medical fixation^6,7^. However, since 2015, the MH started to adopt a different rule in the methodology of allocating doctors, with the description of eight vulnerability profiles based on proportions of census sectors with a population in extreme poverty^8^. In this scenario, the number of physicians enrolled by the PMMB increased from 14,168 to 18,240, and the number of municipalities from 3,785 to 4,028, corresponding to 73% of cities in Brazil^9,10^. In São Paulo, of the 645 municipalities of the state, 385 were participating in the PMMB in 2015, with 2,468 doctors^11^.

The work of Cuban doctors has always been predominant in the project, occupying almost 80% of the vacancies at the beginning of the PMBB and 52% in the last quarter of 2018^12,13^. We emphasize that the order of recruitment and replacement for the municipalities prioritized physicians registered in the CRM, followed by exchange students trained abroad and, only after these two calls, foreign cooperates (mainly Cubans) without diploma revalidation. This shows the importance of the participation of foreign physicians (Cubans and of other nationalities) in the project, representing a clear advance in the coverage of primary care services.

This configuration was to provide a framework for the restructuring, and the challenge for the program is the announcement by the Cuban government on its withdrawal from the MDP in November 2018, which imposes effort on the logistics of the large size of the PAHO/WHO for the return of the physicians to Cuba, as well as a challenge to the Brazilian government, in transition, for the immediate replacement of such a contingent, considering the proven effectiveness of the MDP in the papers about the reduction in hospital admissions for conditions sensitive to primary care, and the increase in the number of queries, visits to households, and to the patient’s satisfaction^14^. With the change in federal management, in 2019, the new rules for the renewal of the contract of the physician participant, and the replacement of the vacancies that have been advertised in the public notices, with the vulnerability of a municipal exclusively to the profiles 4 and 8, so that the other – ratings of 1 to 3, which correspond to the groups of II, III, and IV of the Primary Care Floor (PCF), in addition to the state capitals and metropolitan regions – will not be the professionals and exchanged at the end of the contract, and, therefore, the municipalities will have to engage the physicians in the basic care of their own resources to secure their assistance to the population^17^.

Given this new scenario, the objective of our article is to estimate the financial impact, for the MH and for the municipalities, resulting from the change in the priority rules by vulnerability profile for PMMB replacement in the state of São Paulo, from January 2019 to March 2022.

This is a financial impact analysis study, exempted from authorization by the Research Ethics Committee of the Faculdade de Odontologia de Piracicaba, of the Universidade Estadual de Campinas, by Decree No. 13/2019, since it uses secondary data and available for public consultation. The number of physicians participating in the PMMB between December 2018 and April 2019 was obtained from the official website of the MDP and the *Cadastro Nacional de Estabelecimentos de Saúde* (CNES – National Register of Health Institution).

The perspective of the study was focused on the SUS, including the impact on municipalities and federal management. In this sense, the costs for municipalities and MH related to the implementation of the PMMB and the establishment of the professional in the municipality (comprising payment exchange for the MH, food aid and housing aid by the municipalities) were estimated, and verified how many professionals were effectively replaced from the 11th cycle (each cycle of participation of the doctor lasts for three years). Another estimate was made by projecting the replacement of a professional by the municipality (outside the project), that is, with direct hiring by the municipal management according to the Consolidation of Labor Laws (CLT), with salary equivalent to the net value of the project scholarship, plus labor costs. We adopted the assumption that the municipality would receive, through the federal budget for primary care – the variable Primary Care Floor (PCF)–, resource for the cost of Family Health team (FHS) in the amount of R$ 7,130.00 (in the vast majority of situations in the state of São Paulo) and that, by participating in the PMMB, all units that receive a doctor reduce their federal transfer (PCF variable) to R$ 4,000.00^18^. However, there are exceptions for the variable PCF value received by some units that are in settlement quilombola areas, which receive a slightly higher financial resource, although this is not a reality within the state of São Paulo, and is therefore disregarded as a presupposition.

Another assumption was regarding the amounts of housing and food aid, since the physicians participating in the project receive them as a counterpart of the municipalities to the PMMB, via physical property rental or monetary ressource^19,20^. We assigned a value of R$ 2,000.00, on average, for the two items grouped, within the minimum and maximum limits defined in ordinance.

A spreadsheet for estimating costs was prepared in the Excel tool, with data collected from the following websites and databases:

1. http://www.maismedicos.gov.br – on this site, in the “results” section, we analyze the ordinances with the names of the doctors that joined the program, by cycles, as well as the edicts, to reference the duration of the contract and the amount of the scholarship received by the physician.
2. http://www.maismedicos.gov.br/consulta-por-cidade# – in the tab “query by city,” we obtained the vacancies of the program by municipality of the state of São Paulo.
3. http://cnes.datasus.gov.br – on the website of the CNES, we searched each professional, entering the names of the physicians in the guidelines, to verify if they were still working in the municipalities where they were allocated.
4. http://portalfns.saude.gov.br – the portal of the *Fundo Nacional de Saúde* (FNS – National Health Fund was consulted to verify the values received by the units that had the MDP implanted or not.

Thus, as presented in Box 1, the values were initially accounted for as MH costs, with the exception of losses in the values passed on to municipalities due to incentives for each FHS. From the 9th cycle, that is, from February 2019, at the time of the renewal of the three-year contract of the PMMB physicians, these costs compromised the budgets of the municipalities, because some had no physicians replaced and were or will be forced to hire new professionals, paying their salaries and other charges. However, when this doctor is hired by the municipality and allocated in FHS, there will be a new contribution of financial resources resulting from the increase in the amount passed on by the MH to such teams. These repositions were computed, with each new cycle, until March 2022, when this rule should be exhausted with the closure of the 17th cycle (referring to physicians that joined from 2019). For the purposes of our study, the 17th cycle will be considered the last with new professionals incorporated into the PMMB.

**Box 1:** Methodology for costing:

Methodology for costing
**Ministry of Health:**
Duration of the PMMB: (a) scholarship related to the payment of the professional; and (b) decrease in the transfer values for the eSF.
After non-replenishment: (a) non-payment of scholarship; and (b) increase in the transfer values to the eSF.
**Municipalities**
Duration of the PMMB: (a) money received by the MH for the project; (b) food and rent by the municipalities; and (c) decrease in the transfer values for the eSF.
After non-replenishment: (a) hiring of physicians (market value equal to the value of the project grant) + labor charges; and (b) increase in the transfer values to the FHS.

There was no discount rate because the values were changing each cycle. Moreover, we elaborated a sensitivity analysis (fixed) of 30% for more or less of the total values to be spent by the MH and the municipalities. This is particularly important due to the regional differences within the state, as well as the difficulties of fixing physicians and the offer of more or less attractive salaries depending on the conditions of local infrastructure, cultural characteristics and the proximity of training centers. One last reason is the possible bias related to the assumptions, which can be heterogeneous considering the various variables that modulate the professional’s salary and the attractiveness of fixing in the municipality.

The parameters for estimating the financial impact, described in Box 2, considered the following cost items: cost of scholarships for professionals – “scholarship”; difference in values in the federal financial transfer to the FHS (variable PCF) – “PCF_dif_”; expenditures with references – “Ref.”; PCF variable in unit with a PMMB physician – “PCF_PMMB_”; physician’s wage with employment charges – “Physician wage”; rent – “Rent.”; and feeding – “Food.”

**Box 2:** Parameters for estimating the budget impact: Ministry of Health and municipalities, from 2019 to 2022.

MH investment	Physicians’ scholarship (PS)	Difference in values in the transfer of the variable PCF to the eSF (PCF_dif_)	Expenditure on references (Ref.)*	Variable PCF with PMMB physician (PCF_PMMB_)
	R$ 11,865.60	R$ 3,130.00 (R$ 7,130.00-R$ 4,000.00)	R$ 6,500. 00 (n=1/central) and R$ 6,000. 00 (n= 7/SP decentralized)	R$ 4,000.00
Municipalities’ budget	Medical salary with employment charges (Physician Wage)	Difference in values in the transfer of the variable PCF to the eSF (PCF_dif_)	Rent (Rent.)	Feeding (Food.)
	R$ 20,236.25	R$ 3,130.00	R$ 2,000.00	R$ 600.00

* Decentralized references are those established in the states, and the centralized reference is that established in the Ministry of Health (Brasilia).

Box 3 shows the structure of the predicted estimate for decomposition of the gradual reduction in the number of vacancies of the PMMB:

**Box 3:** Estimate provided for decomposition of the gradual reduction of the number of vacancies of the PMMB.

MH	1st baseline: January 2019 (Ref. + N physicians * Scholarship + N physicians * 4,000) From the 3rd line: March 2019 {Ref. + [N physicians PMMB * scholarship] + [N physicians * PCF_PMMB_] + [(N baseline physicians PMMB – N physicians current month)* PCF_dif_]}
Municipalities	1st baseline: January 2019 {[N physicians * (Food. + Rent.)] + (N doctors * PCF_PMMB_)} From the 3rd line: March 2019 {[N physicians PMMB * (Food. Rent.)] + [(N baseline physicians – N physicians current month] * (Physician wage) – [(N baseline physicians – N physicians current month] * (PCF_PMMB_ + PCF_dif_)}

Box 4 shows the estimate of the amount for hiring a physician in celetist regime and its annual cost. This estimate was based on the hypothesis that the professional receives exactly the same net value of the PMMB scholarship and, thus, possible bias relative to differences within the state is controlled.

**Box 4:** Values attributed to the costs of hiring a celetist physician with net wage aligned to the amount of the scholarship paid by MH.

Gross	R$ 15.809,57
Brazilian National Social Security Institute (INSS)	R$ 642.34
Income Tax (A)	R$ 3,301.63
**Net**	**R$ 11,865.60**
INSS-employer quota (monthly)	R$ 3,161.91
Brazilian Government Severance Indemnity Fund for Employees (monthly)	R$ 1,264.77
Holidays + 1/3	R$ 21,079.43
13th salary	R$ 15,809.57
Notice	R$ 15,809.57
INSS-employer quota (notice)	R$ 3,161.91
FGTS (notice)	R$ 1,264.77
INSS-employer quota (holidays + 1/3)	R$ 4,215.89
FGTS (13th salary)	R$ 1,264.77
**Monthly total**	**R$ 20,236.25**
**Annual total for the employer, already including termination**	**R$ 285,204.64**

## RESULTS

Table 1 describes the assistance, the stratified percentage of dependence of the PMMB in function of the vulnerability described above, obtained from the number of FHS of the municipality and the physicians of the project allocated in them. We found that, of the 373 municipalities, only 86 are classified within the replacement ruler of professionals by the PMMB (profiles 4 to 6 of Table 1). Another important information is that 131 municipalities are dependent on PMMB, which varies from 40% to 100%.

**Table 1:** Number of municipalities by percentage of dependence on PMMB and by stratification of the vulnerability indicator for the state of São Paulo.

Dependence of PMMB in municipalities (%)	Nº of municipalities by Vulnerability Indicator*						
	Types of line						
	1	2	3	4	5	6	Overall Total
0–20	53	39	26	7	1	1	127
20–40	27	50	17	11	4	6	115
40–60	16	36	5	25	-	7	89
60–80	-	6	3	2	-	1	12
80–100	1	8	-	16	-	5	30
**Overall total**	**97**	**139**	**51**	**61**	**5**	**20**	**373**

* Numbers within the indicators from 1 to 3 represent the municipalities that will not have physician replacement, according to the new PMMB criterion.

Figure shows the projection of the number of physicians and municipalities in the state of São Paulo participating in the PMMB without replacement of these professionals (profiles 1 to 3), from January 2019 to March 2022. The state will lose 2,213 doctors, and 287 municipalities will no longer participate in the PMMB because they do not meet the new criterion of the vulnerability scale, representing a reduction of 76.9% of the total number of vacancies in the state in this period. The number of professionals that will remain will be 295 (vulnerability profile > 3).

**Figure:**
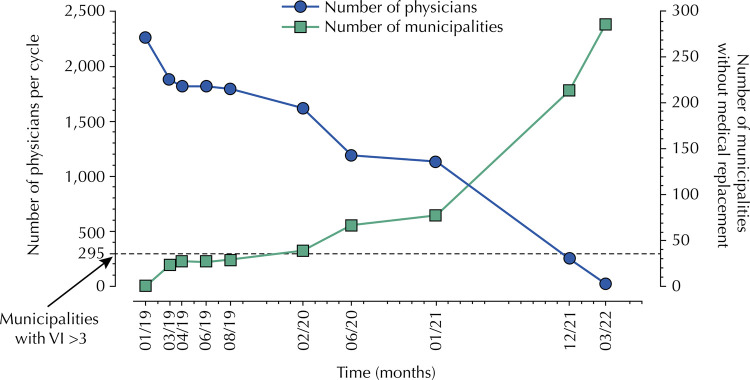
Projection of the number of physicians and municipalities in the state of São Paulo participating in the PMMB without physician replacement (profiles 1 to 3), from January 2019 to March 2022.

VI: vulnerability index.

Table 2 shows the breakdown of the project cycles, which correspond to published notices projecting the physician’s stay in the program for three years – we assume as a fixed effect the assumption that the physician will not close the contract before its completion, although this occurs in at least 20% of cases. In the column of physicians per cycle are projected vacancies within the new criterion, based on the departures already started in 2019 and that will occur over the three years of existing contracts. We started the estimates in January 2019, in São Paulo, with 2,533 physician vacancies and, for 2022, we expect to have 320 program professionals within the state (295 in municipalities with a vulnerability index greater than 3, and another 25 at the end of the contract).

**Table 2:** Financial impact decomposed by cycles, number of physicians and expenditures of federal and municipal entities; and sensitivity analysis with description of more pessimistic and more optimistic scenarios.

	Cycle (month / year)	Number of physicians per cycle	MH (R$)	Municipality (R$)
	MDP ceiling (January/2019)	2.533	40,236,064.80	−8,612,200.00
	9th cycle (vacancies until March/2019)	2.165	35,549,364.00	10,452,099.85
	10th cycle (vacancies until April/2019)	2.107	34,810,699.20	11,061,462.33
	11th cycle (vacancies until June/2019)	2.106	34,797,963.60	11,071,968.58
	12th cycle (vacancies until August/2019)	2.084	34,517,780.40	11,303,106.07
	13th cycle (vacancies until February/2020)	1.909	32,289,050.40	13,141,699.75
	11th cycle (vacancies until June/2019)	1.484	26,876,420.40	17,606,855.83
	15th cycle (vacancies until January/2021)	1.427	26,150,491.20	18,205,712.06
	16th cycle (vacancies until December/2021)	546	14,930,427.60	27,461,717.96
	17th cycle (vacancies until March/2022)	320	12,052,182.00	29,836,130.36
	Total costs (39 months)		1,124,135,514.00	593,612,104.77
	Financial impact on municipalities between January/2019 and March / 2022			−929,487,904.77
Sensitivity analysis	More pessimistic scenario (+30%)			−1,208,334,276.20
	More optimistic scenario (-30%)			650,641,533.34‬
	Financial impact on MH between January/2019 and March/2022			445,071,013.20
Sensitivity analysis	More pessimistic scenario (+30%)			578,592,317.16
	More optimistic scenario (-30%)			311,549,709.24

The economic projections follow the scenario of federal defunding and municipal increase, assuming that the municipality hires physicians to fill the local lack of assistance. In this case, there is a clear reversal of the flow of funding from the MH to the municipalities. The total invested in the 39 months evaluated will be R$ 1.1 billion for the MH and R$ 593.6 million for the municipalities. However, the financial impact considers intrinsic and extrinsic costs, that is, the costs within the project and those arising from the hiring of professionals by the municipalities (on the outside) and the variations of the PCF. In this case, the financial impact for the municipalities that will need to replenish the physicians will be R$ 929,487,904.77 (with sensitivity analysis ranging from R$ 650,641,533.34 to R$ 1,208,334,276.20). This is easily seen when one checks the negative spending (what was stopped spending) of the municipalities in January 2019, increasing from the completion of the cycles, in March and April 2019, and in the cycles of the future months. Obviously, such a projection considers a fixed effect of these costs, assuming the immediate replacement of the professional, which probably will not correspond to reality.

## DISCUSSION

The transversality of public health policies is visible when we look back on the expansion of care in recent years, promoted and induced by the PNAB, since 2006, until its most recent version, 2017. The subject on the agenda has always been the expansion of coverage, the requalification of the units, the improvement of quality indicators and the focus on user care, with integrated and complete staff. To this end, several managers opted to join the PMMB as a way to increase the coverage of local care, maintain medical fixation and encourage the link between users and health teams – then incomplete due to the absence of medical professional^2,20^.

The analysis of the financial impact clearly shows that, with the new weighting of the vulnerability scale, 76.9% of municipalities in the state of São Paulo will lose their PMMB physicians and, thus, they will have to disburse sufficient amount to make the new vacancy attractive to continue to provide the expanded assistance of recent years from their own resources, even with a limited budget. It should be remembered, in this case, the spending ceiling imposed by Constitutional Amendment No. 95/2016 and the Fiscal Responsibility Law (FRL), in addition to the decrease in tax collection since 2015, which creates a possible scenario of instability in the provision of health services, giving space for planning with changes in the care model^21^.

According to Ipea^22^, municipalities had an increase in the allocation of their own resources for SUS financing from 25.4% in 2003 to 31.1% in 2017. In the same period, the states had a small increase in the allocation of resources for health, from 24.5% to 25.7%, whereas that of the Union decreased from 50.1% to 43.2%. This implies budgetary overload for municipalities that need to contemplate all other sectors (education, infrastructure, etc.).

PMMB, in full operation, had a cost considered moderate, according to the indicators presented to the population, could correct distortions of distribution of physicians and led to reflection on the scarcity and medical training^23^. Nevertheless, the challenge for 2019 fell on the tension of hiring links, the lack of assistance, the reorientation of primary care, the underfunding and the change of the care model, in a scenario in which municipalities expanded all their primary care using the PMMB as a driver, with a percentage of dependence greater than 50%^24^.

However, budgetary constraints hinder the full implementation of Public Health Policy. Thus, we find it conflicting to think about the increase in primary care coverage and, at the same time, impose a process of disqualification and restructuring of the PMM, allied to the political pressure of the Medical Corporation for the end of the program and the forces of the private sector, which seeks gaps to seize fractions of the health market^27^. It would be good if important decisions on management strategies such as the PMMB were agreed between the three entities (Municipality, State and Federal government) in a distributive way regarding the weight and responsibilities of health actions. However, we found that the decision to change the rule of replacement of PMMB professionals was vertical and, in the timeline, we realized its impact on the municipalities, disregarding the micropolitics of loco-regional scenarios.

The federal government launched, in May 2019, the Health Program at the hour for medium and large municipalities, which offers financial incentives to Family Health Units (FHU) to extend their hours of service, and may even double the monthly transfer for their cost. In total, 546 FHU (mostly in the South and Southeast regions) were enrolled in the program in August 2019^28^. However, the rules for accreditation are strict, and this may not solve the problem in the short term, given the high cost of hiring professionals.

A provisional measure was recently launched instituting the physicians for Brazil Program, which provides for increased coverage of vulnerable areas, with a new vulnerability scale for municipalities (made by the Brazilian Institute of Geography and Statistics), and guarantee of labor rights to physicians (including hiring by CLT and career progression after two years in the program). However, some parts of the program seem to be controversial, namely: (a) to permit the establishment of an agency of a legal person in private law to manage the new form of the provision; and (b) the selection to the public, only to physicians registered in the CRM, and (c) the total workload of 60 hours of work per week; and (d) the ability of the physician to be a scholar, and, at the end of two years, to make proof of the title of specialist, but have not been trained in residence; and (e) the proof is at the end of the time period for the employment of the celetist scheme by the MH. This last item was not stated in the Law No 13958, which set up the More Doctors for Brazil Program^29^, even though it has been propagated into the reports from the MH, and the question of otherness, not only because it differs from the official discourse is not the creation of more parking spaces to the public for the contests and the selections, and the budget cuts are strict (in the new program, in fact, you should have to spend 40% more when compared with PMMB), but also because of the new physicians will be hired initially for two years, and by the middle of the bag (along the lines of the PMMB), and then have to pass the selection to the public, probably in the year 2022, which is the year of federal elections – in default, therefore, of the law. We suggested that the selection periods should coincide with that previously reported to allow the effective hiring of physicians.

A suggestion to improve the efficiency of the new program would be to transfer the amounts related to hiring by incentives (need to use the amount specifically for the payment of professionals), such as incentives for family health teams, oral health and many others already existing. This would eliminate the high cost that will be created with the maintenance of an administratively complex structure such as the Agência para o Desenvolvimento da Atenção Primária à Saúde (Adaps – Agency for the Development of Primary Health Care), which, in addition to serving as the maintainer of the medical program in Brazil, has a clear objective of allowing greater participation of the private sector.

The limitations of our study relate to the assumptions in the methodology, namely: (a) the establishment of an fixed effect as a consequence of the loss of the replacement of the physician’s in the PMMB, and are hiring immediately for the physician, by the city manager, who in practice may or may not be, since the management might be limited in terms of budget and percent spent on payroll, according to the Fiscal Responsibility Law (FRL); or (b) the assumption that all physicians will end a contract, even though the study shows that at least 20.8% drop out of the job in the first year^30^; and (c) the use of secondary data, in such a way that the information is handed over to the system to which it provides; and (d) the fixing of a salary for employment with the same net present value from the stock exchange in the PMMB, although regional variations can have different scenarios. Thus, given the structural uncertainty, we decided to parameterize the values of the financial impact by elaborating a sensitivity analysis and, therefore, obtaining a range of expenses that may vary according to specific situations.

Finally, we conclude that the change in the prioritization of vulnerability profiles (4-8) adopted for the PMMB result in important consequences, namely lack of assistance for the population and high budget impact for municipalities in the state of São Paulo in a scenario of budget limitations.
