# Supplementary material for: Financial impact of the change in the vulnerability profile of More Doctors Program
Source: Rev Saude Publica. 2020 Dec 4;54:148. doi: 10.11606/s1518-8787.2020054002156 (PMC7726938; doi:10.11606/s1518-8787.2020054002156)
Supplement: Translation

# Translation: Impacto financeiro da alteração do perfil de vulnerabilidade no Programa Mais Médicos

**Authors:** Denise de Fátima Barros Cavalcante, Carolina Vitti Domingues, Diego Roberto Meloni, Frederico Machado de Almeida, Livia Fernandes Probst, Yuri Wanderley Cavalcanti, Marcelo de Castro Meneghim, Antonio Carlos Pereira

**Affiliations:** I Brasil Universidade Estadual de Campinas. Faculdade de Odontologia de Piracicaba. Pós-Doutoranda em Odontologia em Saúde Coletiva. Piracicaba, SP, Brasil; II Brasil Universidade Estadual de Campinas. Faculdade de Odontologia de Piracicaba. Programa de Pós-Graduação em Odontologia. Piracicaba, SP, Brasil; III Brasil Universidade de São Paulo. Faculdade de Medicina de Ribeirão Preto. Programa de Pós-Graduação em Saúde Pública. Ribeirão Preto, SP, Brasil; IV Brasil Faculdade de Ciências Médicas da Santa Casa de São Paulo. São Paulo, SP, Brasil; V Brasil Universidade Federal de Mato Grosso do Sul. Faculdade de Odontologia de Campo Grande. Departamento de Saúde Coletiva. Campo Grande, MS, Brasil; VI Brasil Universidade Federal da Paraíba. Centro de Ciências da Saúde. Departamento de Odontologia Social. Joao Pessoa, PB, Brasil; VII Brasil Universidade Estadual de Campinas. Faculdade de Odontologia de Piracicaba. Departamento de Odontologia Social. Piracicaba, SP, Brasil

**Keywords:** Médicos Graduados Estrangeiros, provisão & distribuição, Consórcios de Saúde, Gastos em Saúde, Avaliação de Programas e Projetos de Saúde, economia

## Abstract

**OBJETIVO:**

Calcular o fluxo de profissionais e o impacto financeiro do Projeto Mais Médicos para o Brasil (PMMB), dentro do Programa Mais Médicos (PMM), para o Ministério da Saúde e os municípios do estado de São Paulo aderentes, no período de janeiro de 2019 a março de 2022.

**MÉTODOS:**

Realizou-se estudo de impacto financeiro a partir de bases de dados secundários públicos do estado de São Paulo. O número de vagas do PMMB por município, de médicos do projeto e os perfis de vulnerabilidade foram descritos para dimensionar a perda de reposição de profissionais no período.

**RESULTADOS:**

No intervalo de tempo especificado, o número de médicos do PMMB em cidades a ele aderentes passará de 2.533 para 320, e o número de municípios participantes de 373 para 86. O impacto orçamentário para os municípios que necessitarão repor médicos será de R$ 929.487.904,77 (com análise de sensibilidade variando de R$ 650.641.533,34 a R$ 1.208.334.276,20).

**CONCLUSÃO:**

A mudança de metodologia da vulnerabilidade adotada para o PMMB trará sérias consequências, ou seja, desassistência da população e alto impacto financeiro para os municípios do estado de São Paulo em um cenário de limitações orçamentárias.

## INTRODUÇÃO

Todo o contexto de mudança do olhar coletivo presente no arcabouço da implantação e ampliação da Política Nacional de Atenção Básica (PNAB), a partir do Sistema Único de Saúde (SUS), trouxe demandas concretas tanto na formação profissional quanto na gestão do trabalho para as políticas de saúde, sendo claramente identificadas no Ministério da Saúde (MS), nas secretarias estaduais e municipais^1,2^.

O SUS corroborou a necessidade de implantar uma nova política que fosse capaz de solucionar a falta de profissionais médicos, o déficit de acesso devido a sua má distribuição, bem como a qualidade da atenção básica, que refletia a carência de melhorias da formação para o serviço público. Seguindo esse direcionamento, uma pesquisa do Instituto de Pesquisa Econômica Aplicada (Ipea), em 2011, revelou a falta de médicos como principal problema do SUS para 58,1% dos brasileiros, enquanto a campanha “Cadê o Médico?”, realizada pela Frente Nacional dos Prefeitos (FNP) em Brasília, em 2013, demonstrou a insatisfação dos gestores com a fixação dos profissionais médicos em seus territórios^3^.

Dessa forma, a implantação do Programa Mais Médicos (PMM) em 2013 (Lei nº 12.871/2013) significou não só a criação de uma política de qualificação e investimento orçamentário do governo federal na atenção básica dos municípios brasileiros, como também o reconhecimento dessas demandas no contexto político institucional. O PMM é composto por três eixos: (a) ampliação e melhoria da infraestrutura de unidades de saúde; (b) provimento emergencial de médicos para áreas desassistidas; e (c) formação de recursos humanos para o SUS^4^. No quesito provimento, havia a adesão de médicos formados em instituições de ensino superior brasileiras e estrangeiras ao Projeto Mais Médicos para o Brasil (PMMB), interesse específico deste estudo.

Desde o início do PPMB, o MS interviu diretamente na provisão para esses municípios pelo chamamento de médicos registrados nos Conselhos Regionais de Medicina (CRM), intercambistas individuais (brasileiros e estrangeiros formados no exterior) e médicos cubanos (estes através de cooperação com a Organização Pan-Americana da Saúde – Opas), de forma que recrutamentos foram realizados em fases, segundo a ordem citada, até o preenchimento de todas as vagas. Assim, o MS foi protagonista na alocação, no pagamento de bolsas, no fomento e na ampliação da mudança da formação médica^5^.

Inicialmente, em 2013, as regras para adesão dos municípios prioritários para o provimento emergencial do PMMB eram mensuradas por indicadores de vulnerabilidade baseados no PIB, na cobertura de planos de saúde, no número de residentes em área rural, na extrema pobreza, no percentual de beneficiários do Bolsa Família, nas horas trabalhadas por médicos na atenção básica, no percentual de leitos por mil habitantes e na fixação médica^6,7^. Contudo, a partir de 2015, o MS passou a adotar uma regra diferente na metodologia de alocação dos médicos, com a descrição de oito perfis de vulnerabilidade baseados em proporções de setores censitários com população em extrema pobreza^8^. Neste cenário, o número de médicos aderidos pelo PMMB aumentou de 14.168 para 18.240, e o número de municípios de 3.785 para 4.028, correspondendo a 73% das cidades do Brasil^9,10^. Em São Paulo, dos 645 municípios que compõem o estado, 385 estavam participando do PMMB em 2015, com 2.468 médicos^11^.

A atuação de médicos cubanos sempre foi predominante no projeto, ocupando quase 80% das vagas no início do PMBB e 52% no último trimestre de 2018^12,13^. Salientamos que a ordem de recrutamento e reposição para os municípios priorizava médicos com registro no CRM, seguidos de intercambistas formados no exterior e, somente após esses dois chamamentos, os cooperados estrangeiros (cubanos, principalmente) sem revalidação de diploma. Isso demonstra a importância da participação de médicos estrangeiros (cubanos e de outras nacionalidades) no projeto, representando um claro avanço na cobertura de serviços da atenção básica.

Tal configuração teve como marco de reestruturação e desafio para o programa o anúncio do governo cubano sobre sua retirada do PMM em novembro de 2018, situação que impôs esforço logístico de grande porte à Opas para retorno dos médicos a Cuba, bem como um desafio ao governo brasileiro, em transição, para a imediata reposição de tal contingente, haja vista a comprovada efetividade do PMM em estudos abordando a redução de internações por condições sensíveis à atenção primária e o aumento do número de consultas, de visitas domiciliares e da satisfação do paciente^14^. Com a mudança de gestão federal em 2019, novos critérios para renovação do contrato de médicos já participantes e para reposição de vagas foram divulgados nos editais, com a vulnerabilidade municipal calibrada exclusivamente para os perfis de 4 a 8, de modo que os demais – das classificações de 1 a 3, correspondentes aos grupos II, III e IV do Piso de Atenção Básica (PAB), além das capitais e regiões metropolitanas – não terão os profissionais substituídos após o término do contrato e, consequentemente, os municípios terão que contratar médicos da atenção básica com recursos próprios para garantir sua assistência à população^17^.

Diante desse novo cenário já estabelecido, o objetivo deste artigo é calcular o impacto financeiro, para o MS e para os municípios, decorrente da mudança nas regras de prioridade por perfil de vulnerabilidade para reposição do PMMB no estado de São Paulo, no período de janeiro de 2019 a março de 2022.

Trata-se de um estudo de análise de impacto financeiro, dispensado de autorização pelo Comitê de Ética em Pesquisa da Faculdade de Odontologia de Piracicaba, da Universidade Estadual de Campinas, por meio do ofício nº 13/2019, uma vez que utiliza dados secundários e disponíveis para consulta pública. O número de médicos participantes do PMMB entre dezembro de 2018 e abril de 2019 foi obtido no site oficial do PMM e no Cadastro Nacional de Estabelecimentos em Saúde (CNES – DataSUS e Sistema de Gerenciamento de Programas).

A perspectiva do estudo foi voltada para o SUS, incluindo o impacto para os municípios e a gestão federal. Nesse sentido, foram calculados os custos para municípios e MS referentes à implantação do PMMB e à fixação do profissional no município (compreendendo bolsa de pagamento pelo MS, auxílio-alimentação e auxílio-moradia por parte dos municípios), e verificados quantos profissionais foram efetivamente repostos a partir do 11º ciclo (cada ciclo de participação do médico tem duração de três anos). Outro cálculo foi realizado projetando a reposição de um profissional por parte do município (fora do projeto), ou seja, com contratação direta pela gestão municipal segundo a Consolidação das Leis do Trabalho (CLT), com salário equivalente ao valor líquido da bolsa do projeto, acrescido dos encargos trabalhistas. Adotamos o pressuposto de que o município receberia, através da verba federal para a atenção básica – o Piso de Atenção Básica (PAB) variável –, recurso para custeio de equipe de Saúde da Família (eSF) no valor de R$ 7.130,00 (na grande maioria das situações no estado de São Paulo) e de que, ao participar do PMMB, todas as unidades que recebem um médico reduzem seu repasse federal (PAB variável) para R$ 4.000,00^18^. Contudo, há exceções para o valor PAB variável recebido por algumas unidades que estão em áreas de assentamento ou quilombolas, as quais recebem recurso financeiro ligeiramente maior, muito embora isso não seja uma realidade dentro do estado de São Paulo, sendo, portanto, desconsiderada enquanto pressuposto.

Outro pressuposto assumido foi a respeito dos valores dos auxílios-moradia e alimentação, pois os médicos participantes do projeto os recebem como contrapartida dos municípios ao PMMB, por meio de aluguel de imóvel físico ou recurso pecuniário^19,20^. Atribuímos um valor de R$ 2.000,00, em média, para os dois itens agrupados, dentro dos limites mínimo e máximo definidos em portaria.

Elaborou-se uma planilha para a estimativa de custos, na ferramenta Excel, com dados colhidos nos seguintes sites e bancos de dados:

1. http://www.maismedicos.gov.br – neste site, na seção “Resultados”, analisamos as portarias com nomes dos médicos que ingressaram no programa, por ciclos, bem como os editais, para referenciar a duração do contrato e o valor da bolsa recebida pelo médico.
2. http://www.maismedicos.gov.br/consulta-por-cidade# – na aba “Consulta por cidade”, obtivemos as vagas do programa por cidade do estado de São Paulo, cenário da pesquisa.
3. http://cnes.datasus.gov.br – no site do cadastro nacional de estabelecimentos de saúde, pesquisamos cada profissional, inserindo os nomes dos médicos constantes nos editais, a fim de verificar se ainda estavam trabalhando nos municípios onde foram lotados.
4. http://portalfns.saude.gov.br – o portal do Fundo Nacional de Saúde foi consultado para verificação dos valores recebidos pelas unidades que tinham o PMM implantado ou não.

Desta forma, como apresentado no Quadro 1, os valores foram contabilizados inicialmente como custos do MS, excetuando-se as perdas nos valores repassados aos municípios em razão dos incentivos para cada eSF. A partir do 9º ciclo, ou seja, a partir de fevereiro de 2019, na ocasião da renovação do contrato de três anos dos médicos do PMMB, esses custos comprometeram os orçamentos dos municípios, pois alguns não tiveram médicos repostos e foram ou serão obrigados a contratar novos profissionais arcando com salários e demais encargos. Todavia, quando esse médico for contratado pelo município e alocado em eSF, haverá novo aporte de recursos financeiros resultante do aumento no valor repassado pelo MS para tais equipes. Essas reposições foram computadas, a cada novo ciclo, até março de 2022, quando essa regra deverá se esgotar com o encerramento do 17º ciclo (referente aos médicos que ingressaram a partir de 2019). Para fins deste estudo, o 17º ciclo será considerado o último com novos profissionais incorporados ao PMMB.

**Quadro 1:** Metodologia para cálculo de custos.

Metodologia para cálculo de custos
**Ministério:**
Vigência do PMMB: (a) bolsa referente ao pagamento do profissional; e (b) diminuição dos valores de repasse para as eSF.
Após a não reposição: (a) não pagamento de bolsa; e (b) aumento dos valores de repasse para as eSF.
**Municípios:**
Vigência do PMMB: (a) verba recebida pelo MS para o projeto; (b) alimentação e aluguel por parte dos municípios; e (c) diminuição nos valores de repasse para as eSF.
Após a não reposição: (a) contratação de médicos (valor de mercado igual ao valor da bolsa do projeto) + encargos trabalhistas; e (b) aumento dos valores de repasse para as eSF.

Não houve aplicação de taxa de desconto pois os valores foram mudando a cada ciclo. Além disso, uma análise de sensibilidade (fixa) de 30% para mais ou para menos dos valores totais a serem gastos pelo MS e pelos municípios foi elaborada. Isso é particularmente importante devido às diferenças regionais dentro do estado, como também às dificuldades de fixação dos médicos e ao oferecimento de salários mais ou menos atraentes dependendo das condições de infraestrutura local, das características culturais e da proximidade de centros de formação. Uma última razão é o possível viés relacionado aos pressupostos, os quais podem ser heterogêneos diante das diversas variáveis que modulam o salário do profissional e os atrativos de fixação no município.

Os parâmetros para o cálculo do impacto financeiro, descritos no Quadro 2, levaram em consideração os seguintes itens de custo: valor das bolsas para os profissionais – “Bolsa”; diferença de valores no repasse financeiro federal para as eSF (PAB variável) – “PAB_dif_”; gastos com referências – “Ref.”; PAB variável em unidade com médico do PMMB – “PAB_PMMB_”; salário médico com encargos empregatícios – “Sal. Médico”; aluguel – “Alu.”; e alimentação – “Alim.”.

**Quadro 2:** Parâmetros para cálculo do impacto orçamentário: Ministério da Saúde e municípios, de 2019 a 2022.

Investimento do MS	Bolsas dos médicos (BM)	Diferença de valores no repasse do PAB variável para as eSF (PAB_dif_)	Gastos com referências (Ref.)*	PAB variável com médico do PMMB (PAB_PMMB_)
	R$ 11.865,60	R$ 3.130,00 (R$ 7.130,00-R$ 4.000,00)	R$ 6.500,00 (n=1/central) e R$ 6.000,00 (n=7/SP descentralizadas)	R$ 4.000,00
Orçamento dos municípios	Salário médico com encargos empregatícios (Sal. Médico)	Diferença de valores no repasse do PAB variável para as eSF (PAB_dif_)	Aluguel (Alu.)	Alimentação (Alim.)
	R$ 20.236,25	R$ 3.130,00	R$ 2.000,00	R$ 600,00

* Referências descentralizadas são aquelas estabelecidas nos estados, e a referência centralizada é a estabelecida no Ministério da Saúde (Brasília).

A estrutura do cálculo previsto para decomposição da redução gradual do número de vagas do PMMB está descrita no Quadro 3:

**Quadro 3:** Cálculo previsto para decomposição da redução gradual do número de vagas do PMMB.

MS	1ª linha base: janeiro de 2019 (Ref. + N médicos * Bolsa + N médicos * 4.000) A partir da 3ª linha: março de 2019 {Ref. + [N médicos PMMB * Bolsa] + [N médicos * PAB_PMMB_] + [(N médicos base PMMB – N médicos mês vigor) * PAB_dif_]}
Municípios	1ª linha base: janeiro de 2019 {[N médicos * (Alim. + Alu.)] + (N médicos * PAB_PMMB_)} A partir da 3ª linha: março de 2019 {[N médicos PMMB * (Alim. Alu.)] + [(N médicos base – N médicos mês vigor] * (Sal. Médico) – [(N médicos base – N médicos mês vigor] * (PAB_PMMB_ + PAB_dif_)}

O cálculo do valor para contratação de um médico em regime celetista é apresentado no Quadro 4, bem como seu custo anual. Esse cálculo foi baseado na hipótese de que o profissional receba exatamente o mesmo valor líquido da bolsa do PMMB e, dessa forma, controla-se possível viés relativo às diferenças dentro do estado.

**Quadro 4:** Valores atribuídos aos custos de contratação celetista de um médico com salário líquido alinhado ao valor da bolsa paga pelo MS.

Bruto	R$ 15.809,57
Instituto Nacional do Seguro Social (INSS)	R$ 642,34
Imposto de Renda (IR)	R$ 3.301,63
**Líquido**	**R$ 11.865,60**
INSS – cota patronal (mensal)	R$ 3.161,91
Fundo de Garantia do Tempo de Serviço (FGTS) (mensal)	R$ 1.264,77
Férias + 1/3	R$ 21.079,43
13º salário	R$ 15.809,57
Aviso prévio	R$ 15.809,57
INSS – cota patronal (aviso prévio)	R$ 3.161,91
FGTS (aviso prévio)	R$ 1.264,77
INSS – cota patronal (férias + 1/3)	R$ 4.215,89
FGTS (13º salário)	R$ 1.264,77
**Total mensal**	**R$ 20.236,25**
**Total anual para o empregador, já inclusa a rescisão**	**R$ 285.204,64**

## RESULTADOS

A Tabela 1 descreve, quanto à assistência, o percentual estratificado de dependência do PMMB em relação à vulnerabilidade descrita anteriormente, obtida a partir do número de eSF do município e de médicos do projeto nelas inseridos. Verifica-se que, dos 373 municípios, somente 86 estão classificados dentro da régua de reposição dos profissionais pelo PMMB (perfis 4 a 6 da Tabela 1). Outra informação importante é que 131 municípios apresentam dependência do PMMB, a qual varia de 40% a 100%.

**Tabela 1:** Número de municípios por percentual de dependência do PMMB e por estratificação do indicador de vulnerabilidade para o estado de São Paulo.

Dependência do PMMB nos municípios (%)	Nº de municípios por indicador de vulnerabilidade*						
	Rótulos de linha						
	1	2	3	4	5	6	Total geral
0–20	53	39	26	7	1	1	127
20–40	27	50	17	11	4	6	115
40–60	16	36	5	25	-	7	89
60–80	-	6	3	2	-	1	12
80–100	1	8	-	16	-	5	30
**Total geral**	**97**	**139**	**51**	**61**	**5**	**20**	**373**

* Números dentro dos indicadores de 1 a 3 representam os municípios que não terão reposição de médicos, segundo o novo critério do PMMB.

No Gráfico 1 apresenta-se a projeção do número de médicos e municípios do estado de São Paulo participantes do PMMB sem reposição desses profissionais (perfis 1 a 3), de janeiro de 2019 a março de 2022. Percebe-se que, ao longo do tempo, o estado irá perder 2.213 médicos, e 287 municípios deixarão de participar do PMMB por não preencherem o novo critério da escala de vulnerabilidade, representando uma redução de 76,9% do total de vagas do estado nesse período. O número de profissionais que permanecerão será de 295 (perfil de vulnerabilidade > 3).

**Gráfico 1:** Projeção do número de médicos e de municípios do estado de São Paulo participantes do PMMB sem reposição (perfis 1 a 3), de janeiro de 2019 a março de 2022.

IV: índice de vulnerabilidade.

Na Tabela 2 apresentamos a decomposição dos ciclos do projeto, os quais correspondem a editais publicados com projeção de permanência do médico no programa por três anos – assumimos como efeito fixo o pressuposto de que o médico não encerrará o contrato antes da sua finalização, embora isso ocorra em pelo menos 20% dos casos. Na coluna de médicos por ciclo são projetadas as vagas dentro do novo critério, a partir das saídas já iniciadas em 2019 e que ocorrerão ao longo dos três anos de contratos já existentes. Iniciamos as estimativas em janeiro de 2019, em São Paulo, com 2.533 vagas de médicos e, para 2022, prevemos contar com 320 profissionais do projeto dentro do estado (295 nos municípios com índice de vulnerabilidade maior que 3, e outros 25 em final de contrato).

**Tabela 2:** Impacto financeiro decomposto por ciclos, número de médicos e dispêndios dos entes federal e municipal; e análise de sensibilidade com descrição de cenário mais pessimista e mais otimista.

	Ciclo (mês/ano)	Nº de médicos por ciclo	MS (R$)	Município (R$)
	Teto do PMM (janeiro/2019)	2.533	40.236.064,80	−8.612.200,00
	9º ciclo (vagas até março/2019)	2.165	35.549.364,00	10.452.099,85
	10º ciclo (vagas até abril/2019)	2.107	34.810.699,20	11.061.462,33
	11º ciclo (vagas até junho/2019)	2.106	34.797.963,60	11.071.968,58
	12º ciclo (vagas até agosto/2019)	2.084	34.517.780,40	11.303.106,07
	13º ciclo (vagas até fevereiro/2020)	1.909	32.289.050,40	13.141.699,75
	14º ciclo (vagas até junho/2020)	1.484	26.876.420,40	17.606.855,83
	15º ciclo (vagas até janeiro/2021)	1.427	26.150.491,20	18.205.712,06
	16º ciclo (vagas até dezembro/2021)	546	14.930.427,60	27.461.717,96
	17º ciclo (vagas até março/2022)	320	12.052.182,00	29.836.130,36
	Total de custos (39 meses)		1.124.135.514,00	593.612.104,77
	Impacto financeiro nos municípios entre janeiro/2019 e março/2022			−929.487.904,77
Análise de sensibilidade	Cenário mais pessimista (+30%)			−1.208.334.276,20
	Cenário mais otimista (−30%)			650.641.533,34
	Impacto Financeiro no MS entre janeiro/2019 e março/2022			445.071.013,20
Análise de sensibilidade	Cenário mais pessimista (+30%)			578.592.317,16
	Cenário mais otimista (−30%)			311.549.709,24

As projeções econômicas acompanham o cenário de desfinanciamento federal e incremento municipal, pressupondo-se que o município contrate médicos para suprir a desassistência local. Nesse caso, há uma clara inversão do fluxo de financiamento do MS para os municípios. O total investido nos 39 meses avaliados será de R$ 1,1 bilhão para o MS e R$ 593,6 milhões para os municípios. Contudo, o impacto financeiro leva em conta custos intrínsecos e extrínsecos, ou seja, os custos de dentro do projeto e aqueles oriundos das contratações de profissionais por parte dos municípios (por fora) e das variações do PAB. Nesse caso, o impacto financeiro para os municípios que precisarão repor os médicos será de R$ 929.487.904,77 (com análise de sensibilidade variando de R$ 650.641.533,34 a R$ 1.208.334.276,20). Isso é facilmente visto quando se verifica o gasto negativo (o que se deixou de gastar) dos municípios em janeiro de 2019, aumentando a partir da finalização dos ciclos, em março e abril de 2019, e nos ciclos dos meses futuros. Obviamente, tal projeção considera um efeito fixo desses custos, pressupondo a imediata reposição do profissional, o que provavelmente não corresponderá à realidade.

## DISCUSSÃO

A transversalidade das políticas públicas em saúde é visível quando lançamos um olhar retrospectivo sobre a ampliação da assistência nos últimos anos, fomentada e induzida por meio da PNAB, desde 2006, até sua versão mais recente, de 2017. O assunto em pauta sempre foi a ampliação da cobertura, a requalificação das unidades, a melhoria dos indicadores de qualidade e o foco no cuidado do usuário, com equipe integrada e completa. Com esse objetivo, diversos gestores optaram pela adesão ao PMMB como forma de aumentar a cobertura da assistência local, manter a fixação médica e incentivar o vínculo entre usuário e equipes de saúde – então incompletas devido à ausência do profissional médico^2,20^.

A análise do impacto financeiro nos mostra claramente que, com a nova ponderação da escala de vulnerabilidade, 76,9% dos municípios do estado de São Paulo perderão seus médicos do PMMB e, desta forma, para continuar a prestar a assistência ampliada dos últimos anos, terão que desembolsar, dos próprios recursos, quantia suficiente para tornar a nova vaga atrativa, mesmo tendo orçamento limitado. Deve-se lembrar, neste caso, o teto de gastos imposto pela Emenda Constitucional nº 95/2016 e pela Lei de Responsabilidade Fiscal (LRF), além da diminuição na arrecadação de impostos desde 2015, o que cria um possível cenário de instabilidade no oferecimento de serviços de saúde, dando margem para um planejamento com mudanças do modelo de assistência^21^.

De acordo com o Ipea^22^, os municípios tiveram aumento na alocação de recursos próprios para o financiamento do SUS de 25,4%, em 2003, para 31,1%, em 2017. No mesmo período, os estados tiveram um pequeno aumento na alocação de recursos para a saúde, de 24,5% para 25,7%, enquanto a da União diminuiu de 50,1% para 43,2%. Isso implica sobrecarga orçamentária para os municípios que precisam contemplar todos os outros setores (educação, infraestrutura etc.).

É importante frisar que o PMMB, em pleno funcionamento, teve um custo considerado moderado, segundo os indicadores apresentados à população, conseguiu corrigir distorções de distribuição dos médicos, bem como levou à reflexão sobre a escassez e a formação médica^23^. Não obstante, o desafio para 2019 recaiu sobre o tensionamento dos vínculos de contratação, a desassistência, a reorientação da atenção primária, o subfinanciamento e a mudança do modelo de assistência, num cenário em que municípios ampliaram toda sua atenção básica utilizando como propulsor o PMMB, com percentual de dependência superior a 50%^24^.

No entanto, as limitações orçamentárias impõem obstáculos para a efetivação plena da política pública de saúde. Dessa forma, nos parece conflitante pensar no aumento da cobertura da atenção primária e, ao mesmo tempo, impor um processo de desqualificação e desestruturação do PMM, aliado à pressão política da corporação médica pelo fim do programa e às forças do setor privado, que busca brechas para se apoderar de frações do mercado da saúde^27^. Seria de bom tom que decisões importantes sobre estratégias de gestão como o PMMB fossem pactuadas entre os três entes (Município, Estado e Governo Federal) de forma distributiva quanto ao peso e às responsabilidades das ações em saúde. Contudo, verificamos que a decisão de mudar a regra de reposição de profissionais do PMMB foi vertical e, na linha do tempo, percebemos seu impacto sobre os municípios, desconsiderando a micropolítica dos cenários loco-regionais.

Deve-se salientar que o governo federal lançou, em maio de 2019, o Programa Saúde na Hora para municípios de médio e grande porte, o qual oferece incentivos financeiros a Unidades de Saúde da Família (USF) para estenderem seu horário de atendimento, podendo até dobrar o repasse mensal para seu custeio. Um total de 546 USF (majoritariamente nas regiões Sul e Sudeste) estavam cadastradas no programa em agosto de 2019^28^. Contudo, as regras para credenciamento são restritas, e isso possivelmente não resolverá o problema em curto prazo, em vista do alto custo de contratação dos profissionais.

Cabe ressaltar, também, que recentemente foi lançada medida provisória instituindo o Programa Médicos pelo Brasil, que prevê aumento da cobertura de áreas vulneráveis, com nova escala de vulnerabilidade para os municípios (feita pelo Instituto Brasileiro de Geografia e Estatística), e garantia dos direitos trabalhistas aos médicos (com contratação pela CLT e progressão de carreira após dois anos no programa). Todavia, alguns pontos do programa parecem polêmicos: (a) a autorização da criação de uma agência de pessoa jurídica de direito privado para gerir o novo formato do provimento; (b) a seleção pública apenas para médicos com registro no CRM; (c) a carga horária de 60 horas semanais de trabalho; (d) a possibilidade de o médico ser bolsista e, ao final de dois anos, realizar prova de título de especialista, mas sem formação em residência; e (e) a prova ao final deste período para possível contratação em regime celetista pelo MS. Este último item não consta na Lei 13958 que instituiu o Programa Mais Médicos pelo Brasil^29^, embora tenha sido propagado em informes do MS, e causa estranheza não só porque se distancia do discurso oficial de não criação de novas vagas públicas por concursos/seleções e de contingenciamentos orçamentários severos (o novo programa, aliás, deve ter gasto 40% maior quando comparado ao PMMB), mas também porque os novos médicos serão contratados inicialmente por dois anos, por meio de bolsa (nos moldes do PMMB), e depois deverão passar por seleção pública, provavelmente em 2022, que é ano de eleições federais – descumprindo, portanto, a legislação. Sugere-se que os períodos de seleção não coincidam com o relatado anteriormente a fim de permitir a efetiva contratação dos médicos.

Uma sugestão para melhorar a eficiência do novo programa seria transferir os valores referentes à contratação por meio de incentivos com chancela (necessidade de usar o valor especificamente para o pagamento dos profissionais), nos moldes dos incentivos para equipes de Saúde da Família, Saúde Bucal e muitos outros já existentes. Isso eliminaria o alto custo que será criado com a manutenção de uma estrutura administrativamente complexa como a Agência para o Desenvolvimento da Atenção Primária à Saúde (Adaps), a qual, além de servir como mantenedora do Programa Médicos pelo Brasil, tem objetivo claro de permitir maior participação do setor privado.

As limitações deste estudo estão relacionadas aos pressupostos assumidos em sua metodologia, a saber: (a) o estabelecimento de efeito fixo da consequência da perda de reposição do médico no PMMB e da contratação imediata do médico pelo gestor municipal, o que na prática pode não acontecer, visto que a gestão pode estar limitada em termos orçamentários e pelo percentual gasto com folha de pagamento, conforme a LRF; (b) o pressuposto de que todos os médicos concluirão seus contratos, apesar de estudo mostrar que pelo menos 20,8% deles abandonam o cargo no primeiro ano de atuação^30^; (c) a utilização de dados secundários, de forma que as informações são confiadas ao sistema que as fornece; e (d) a fixação de um salário para contratação com o mesmo valor líquido da bolsa do PMMB, ainda que as variações regionais possam ter cenários distintos. Deste modo, diante da incerteza estrutural, decidimos parametrizar os valores do impacto financeiro elaborando uma análise de sensibilidade e, portanto, obtendo um intervalo de gastos que podem variar conforme situações específicas.

Por fim, conclui-se que a mudança na priorização dos perfis de vulnerabilidade (4–8) adotada para o PMMB trará consequências importantes para a desassistência da população e alto impacto orçamentário para municípios do estado de São Paulo em um cenário de limitações orçamentárias.
